# Muscle-Derived Lumican Stimulates Bone Formation via Integrin α2β1 and the Downstream ERK Signal

**DOI:** 10.3389/fcell.2020.565826

**Published:** 2020-11-06

**Authors:** Jin Young Lee, So Jeong Park, Da Ae Kim, Seung Hun Lee, Jung-Min Koh, Beom-Jun Kim

**Affiliations:** ^1^Asan Institute for Life Sciences, Asan Medical Center, University of Ulsan College of Medicine, Seoul, South Korea; ^2^Division of Endocrinology and Metabolism, Asan Medical Center, University of Ulsan College of Medicine, Seoul, South Korea

**Keywords:** lumican, osteoblast, bone formation, integrin α2β1, ERK

## Abstract

Skeletal muscle and bone are highly interrelated, and previous proteomic analyses suggest that lumican is one of muscle-derived factors. To further understand the role of lumican as a myokine affecting adjacent bone metabolism, we investigated the effects of lumican on osteoblast biology. Lumican expression was significantly higher in the cell lysates and conditioned media (CM) of myotubes than those of undifferentiated myoblasts, and the known anabolic effects of myotube CM on osteoblasts were reduced by excluding lumican from the CM. Lumican stimulated preosteoblast viability and differentiation, resulting in increased calvaria bone formation. The expression of osteoblast differentiation markers was consistently increased by lumican. Lumican increased the phosphorylation of ERK, whereas ERK inhibitors completely reversed lumican-mediated stimulation of *Runx2* and ALP activities in osteoblasts. Results of a binding ELISA experiment in osteoblasts show that transmembrane integrin α2β1 directly interacted with lumican, and an integrin α2β1 inhibitor attenuated the stimulation of ERK and ALP activities by lumican. Taken together, the results indicate that muscle-derived lumican stimulates bone formation via integrin α2β1 and the downstream ERK signal, indicating that this is a potential therapeutic target for metabolic bone diseases.

## Introduction

Bone and skeletal muscle contribute the largest amount of tissues in a lean individual; both respond to physical activity and play a role in protecting internal organs (Tagliaferri et al., 2015). Accumulating clinical evidence indicates that bone and muscle health are highly interrelated, working together throughout an individual’s lifetime (Hirschfeld et al., 2017; Bettis et al., 2018). For example, concomitant losses in bone and muscle mass are frequently observed in older adults (Verschueren et al., 2013; Kim et al., 2015). Both osteoporosis and sarcopenia contribute to the development of fragility fractures that contribute to high disability and mortality (Yu et al., 2014). Thus, effective measures to prevent fractures require continuous efforts to understand the mechanisms underlying bone-muscle crosstalk.

Traditionally, the parallel changes of bone and muscle have been explained by the mechanical force transduction generated by muscle contraction to the adjacent bone (Burr et al., 2002; Qin et al., 2010; Hong and Kim, 2018). Interestingly, aside from being a component of the locomotor system, skeletal muscle is acknowledged as a secretory organ (Pedersen and Febbraio, 2012), and thus it has been hypothesized that muscle-derived factors called “myokines” act as a paracrine signal to regulate bone homeostasis (Hamrick et al., 2010; Kaji, 2014; Giudice and Taylor, 2017). This possibility has been supported in a murine model showing that muscle flaps, compared with other tissues, effectively accelerates fracture healing (Harry et al., 2008, 2009). In addition, recent experiments show that conditioned media (CM) collected from myotubes significantly increase bone formation, suggesting that muscles play a dominant role in releasing bone anabolic factors (Lee et al., 2019).

Evidence is increasing for the importance of biochemical communication between skeletal muscle and bones as well as with other organs, such as brain, pancreas, liver, and adipose tissue (Giudice and Taylor, 2017; Romagnoli et al., 2019). This has led to efforts to identify specific muscle-secreting factors. For example, Norheim et al. (2011) performed, via database searches, proteomic analyses on the CM from cultured human myotubes, and identified 17 novel proteins with secretory features. Among these factors, we are particularly interested in lumican, because it has been consistently detected in the myotube CM of all donors and its expression is markedly increased in skeletal muscle after strength training (Norheim et al., 2011). Importantly, several experiments indicate that lumican may affect the integrin and ERK signaling (Seomun and Joo, 2008; Brezillon et al., 2013), which are key pathways in bone metabolism. These backgrounds raise the possibility that lumican could act as a myokine on adjacent bone homeostasis. To examine this hypothesis, we tested its effects on osteoblast biology through *in vitro* and animal experiments.

## Materials and Methods

### Cell Culture

Mouse C2C12 myoblasts were purchased from the American Type Culture Collection (ATCC, Manassas, VA, United States) and maintained in DMEM supplemented with 10% FBS, 20 mM HEPES, 2 mM L-glutamine, and antibiotics (Life Technologies Corp., Carlsbad, CA, United States) at 37°C in a humidified atmosphere containing 5% CO_2_. CM collected after further incubation for 24 h in serum- and phenol red-free media was regarded as myoblast CM. To induce myogenic differentiation, cells were grown to 90% confluency in maintenance media and then switched to differentiation media (DMEM with 2% horse serum) and cultured for 3 days. Myotube CM was again collected after further incubation for 24 h in serum- and phenol red-free media (Lee et al., 2019). All collected CM was filtered through a 0.45 μm membrane filter and precipitated by lyophilization.

Murine preosteoblast MC3T3-E1 cells (ATCC) were cultured at 37°C in α-minimum essential medium (α-MEM) containing 10% FBS, 100 U/mL penicillin, and 100 μg/mL streptomycin in a humidified atmosphere with 5% CO_2_. The media was changed every 2–3 days. Upon reaching 80% confluence, cells were subcultured with trypsin-EDTA (Gibco, Grand Island, NY, United States). The cells were differentiated into osteoblasts with 50 μg/mL ascorbic acid and 10 mM β-glycerophosphate (Sigma-Aldrich, St. Louis, MO, United States) for 7 days. Human fetal osteoblastic (hFOB) cells were cultured at 33°C in 1:1 mixture of Ham’s F12 Medium Dulbecco’s Modified Eagle’s Medium (DME/F-12 1:1, Hyclone, Logan, UT, United States) containing 10% FBS, 100 U/mL penicillin, and 100 μg/mL streptomycin in a humidified atmosphere with 5% CO_2_.

### Western Blot Analysis

Cells were lysed in RIPA buffer [50 mM Tris–HCl (pH 7.4), 150 mM NaCl, 1% Triton X-100, 1 mM EDTA, 1 mM EGTA, 0.1% SDS, 1% sodium deoxycholate, 1 mM Na3VO4, 1 mM NaF, 1 mM PMSF, and protease inhibitor cocktail]. After a 30-min incubation on ice, lysates were centrifuged at 14,000 rpm for 20 min at 4°C. The protein concentration was measured with a BCA protein assay kit (Pierce Chemical Co., Rockford, IL, United States). Protein samples were separated by SDS-PAGE and transferred to a polyvinylidene fluoride (PVDF) membrane followed by immunoblotting with antibodies (Park et al., 2019). The primary antibodies are as follows: lumican (ab168348) and myogenin (Myog; ab1845) purchased from Abcam (Cambridge, MA, United States); phospho-ERK (9101), phospho-JNK (9251), phospho-AKT (9271), phospho-FAK (Tyr397, 3283), phosphor-Src (Tyr416, 2101), ERK (9102), JNK (9252), AKT (9272), SMAD4 (38454), and integrin β1 (34971) purchased from Cell Signaling Technology (Beverly, MA, United States); integrin α2 (sc-74466); and troponin-C (sc-48347) purchased from Santa Cruz Biotechnology (Dallas, TX, United States); α-tubulin (T9026), β-actin (A3894), and myosin heavy chain (MyHC; M1570) purchased from Sigma-Aldrich.

### Immunofluorescence

C2C12 myoblasts and myotubes were fixed in 4% paraformaldehyde (PFA) for 10 min, washed twice with PBS, permeabilized in 0.01 M sodium citrate buffer containing 0.1% Triton X-100 for 10 min, and washed twice with PBS (Kim et al., 2018). Next, cells were blocked with 2% bovine serum albumin (BSA) in PBS for 1 h. Primary antibodies, such as anti-MyHC (MF20; Developmental Studies Hybridoma Bank, Iowa City, IA, United States) and anti-lumican (Abcam, 1:1000 dilution), were incubated at 4°C overnight. Cells were incubated with Alexa Flour 555-conjugated secondary antibodies (Cell Signaling, 1:1000 dilution) for 1 h, then washed with PBST (0.1% Tween-20 in PBS). Then, the cells were incubated with 4′, 6′-diamidino-2-phenyindole (DAPI; Sigma-Aldrich, 1:10000 dilution) for 2 min and washed with PBS. The samples were mounted using Fluoromount G (Southern Biotech, Birmingham, AL, United States) and images were obtained using a fluorescence microscope (Carl Zeiss, Jena, Germany).

The hFOB cells were washed three times with PBS after treatment with 40 nM 6X His-tagged lumican (R&D systems Inc., Minneapolis, MN, United States) for 30 min. Then cells were fixed with 4% PFA for 10 min, permeabilized with 0.1% Triton X-100 in PBS for 10 min at room temperature. After blocking with 1% BSA in PBS for 60 min, cells were incubated with anti-6X His (Abcam) and anti-integrin α2 and β1 (Abcam) for 16 h at 4°C. Cells were washed with PBS and incubated with Alexa Fluor 488-conjugated and Alexa Fluor 555-conjugated secondary antibodies for 1 h at room temperature. Nuclei were counterstained with DAPI. The stained cells were mounted, and images were obtained using an LSM 710 confocal laser scanning microscope (Carl Zeiss).

### Quantitative Reverse-Transcription PCR

Total RNA was extracted using TRIzol reagent (Invitrogen, Carlsbad, CA, United States) according to the manufacturer’s protocol. After first-strand cDNA synthesis with the Superscript III First-Strand Synthesis System (Invitrogen) using oligo dT primers, Quantitative Reverse-Transcription PCR (qRT-PCR) was performed in triplicate on a Light Cycler^®^ 480 SYBR Green I Master (Roche, Mannheim, Germany; Kim et al., 2018). The primers for *lumican* (NM_008524), *Myog* (NM_031189), *Runt-related transcription factor 2* (*Runx2*; NM_001146038), *osterix* (*Osx*; AY803733), *collagen type 1* α (*Col1*α; NM_007742), *alkaline phosphatase* (*Alp*; NM_007431), *osteocalcin* (*Ocn*; NM_007541), *integrin α2* (*Itgα2*; NM_008396.3), and *integrin β1* (*Itgβ1*; NM_010578) were obtained from Applied Biosystems (Foster City, CA, United States). The threshold cycle (Ct) value for each gene was normalized to the Ct value of the 18S rRNA (NR_003278.3).

### Lumican Silencing Using Short Hairpin RNA

The mouse *lumican* short hairpin RNA (shRNA; 5′-CCT GGA AAC TCG TTT AAT ATA-3′) was constructed using the pLKO.1-*lumican* shRNA cloning vector (Sigma-Aldrich). To knockdown lumican, 70–80% confluent C2C12 cells were infected with control *lumican* shRNA CM or control shRNA CM in the presence of 8 μg/mL polybrene for 6 h. The cells were then washed with PBS and placed in growth media. After incubation for 48 h, infected myoblasts were differentiated into myotubes, and the CM was collected in the same manner as described above.

### Viability Assay

Cell viability was measured using the Cell Counting Kit-8 (CCK-8; Dojindo, Kumamoto, Japan) according to the manufacturer’s instructions. Briefly, 10 μL of WST-8 dye [2-(2-methoxy-4-nitrophenyl)-3-(4-nitrophenyl)-5-(2,4-disulfophenyl)-2H-tetrazolium, monosodium salt] was added to each well of a 96-well plate (Lee et al., 2020). The suspension was incubated for 1 h, and the absorbance was then read at 450 nm with a reference wavelength of 650 nm using a microplate reader (SPECTRAmax 340PC; Molecular Devices, Palo Alto, CA, United States).

### Migration Assay

The chemotaxis assay was performed in a Boyden chamber system using a transwell with an 8-μm-pore size polycarbonate membrane (Corning, NY, United States). Cells were seeded onto the inner chamber at a density of 1.0 × 10^5^ cells/100 μL in α-MEM with FBS for 6 h and then exposed to lumican in the outer chamber for an additional 24 h (Park et al., 2019). The cells on the upper membrane were then completely removed by wiping with a cotton swab, while cells on the lower surface of the membrane were fixed in 4% PFA, stained with crystal violet, photographed, and counted under a dissecting microscope (Carl Zeiss).

### ALP Activity and Osteocalcin Secretion Assays

MC3T3-E1 cells were seeded in 12-well plates at a density of 5 × 10^4^ cells/well and then allowed to differentiate into osteoblasts for 7 days. Cells were then washed with PBS, and ALP activity was measured using the *p*-nitrophenyl phosphate hydrolysis method (Sabokbar et al., 1994). ALP activity was normalized relative to the corresponding total cellular protein content, which was determined by the BCA assay (Pierce Chemical Co.). Osteocalcin concentration in the culture media was measured using an osteocalcin ELISA kit (BT-470; Alfa Aesar, Ward Hill, MA, United States; Sabokbar et al., 1994).

### Luciferase Activity Assay

MC3T3-E1 cells were seeded in 24-well plates at a density of 3 × 10^4^ cells/well and cells were transfected with 100 ng of pGL3 vector and 100 ng of *Runx2* (6xOSE) luciferase reporter plasmid using Lipofectamine 2000 (Invitrogen; Kim et al., 2018). After 6 h, transfected cells were washed and growth medium was added to the plate. The next day, cells were treated with lumican or ERK inhibitors (PD98059 and U0126; Cell Signaling Technology) and allowed to differentiate into osteoblasts. After 3 days, cells were washed with PBS and lysed in 150 μl of passive lysis buffer. Activation of *Runx2* was confirmed using a Dual-Luciferase^®^ Reporter Assay System (Promega, Madison, WI, United States), and luciferase activity was measured using a microplate luminometer (MicroLumat Plus LB96V; Berthold Technologies, Oak Ridge, TN, United States). Relative activity was calculated as the ratio of the firefly reporter to the Renilla luciferase control (pRL-SV40).

### Ligand and Receptor Binding Assay

Lumican was coated onto the wells of Maxisorp 96-well microtiter plates and incubated for 18 h at 4°C. Each well was washed three times with PBST (0.2% Tween-20 in PBS), then the plates were blocked with 1% BSA in PBST for 2 h. Cell lysates were added to the plates, incubated for 2 h, then the wells were washed three times (Kim et al., 2018; Park et al., 2019). Preparations of integrin α2 (Santa Cruz Biotechnology) or β1 antibody (Cell Signaling Technology) in blocking solution were added to the plates and allowed to react for 2 h. After washing, HRP-linked antibody (Cell signaling Technology) was added and the lysates were incubated for 2 h, and then washed five times. The reaction was developed with 100 μl TMB substrate solution and stopped with 100 μl of 1 N H_2_SO_4_. Microtiter plates were measured at 450 nm using a microplate reader (Infinite 200 PRO, Tecan Life Sciences, Zurich, Switzerland).

### *In vivo* Calvaria Bone Formation

Five-week-old male C57BL/6 mice (Orientbio, Seongnam, South Korea) were used in this study. Recombinant lumican (200 μg/kg) or PBS was subcutaneously injected with a 31-gage needle into the right or left parietal bones, respectively, 3 times a week for 4 weeks. Mice were sacrificed 1 week following treatment, and calvaria bones were fixed in 4% PFA for 24 h and decalcified in 0.5 M EDTA in PBS for 2–4 weeks. Decalcified specimens were embedded in paraffin and then coronally sectioned at a thickness of 6 μm. After deparaffinization, sections were rehydrated and then stained with either hematoxylin and eosin (H&E, Sigma-Aldrich) following the manufacturer’s instructions. Using Image-Pro Plus software (Media Cybernetics, Rockville, MD, United States), bone widths were measured at 5 adjacent locations that were at the same distance from the midline between the sagittal suture and the site of muscle attachment, and their mean values were calculated (Lee et al., 2020). To count the number of osteoblasts, the rehydrated sections were stained with toluidine blue O solution (Sigma-Aldrich), and images of the stained osteoblasts were captured using cellSens Standard BX53 software (Olympus, Tokyo, Japan). All methods for animal care and experimental procedures were reviewed and approved by the Institutional Animal Care and Use Committee of the Asan Institute for Life Sciences. The committee abides by the institute of Laboratory Animal Resources (ILAR) guide. All experiments were done, according with the Korean Ministry of Food and Drug Safety (MFDS) guidelines.

### *Ex vivo* Culture

The calvaria bone of timed-pregnant ICR mice (Orientbio) at E21.0 was cut in half along the calvaria sagittal line to include the frontal, coronal, and lambdoid sutures. The bone was placed in a transwell with an 8-μm-pore size polycarbonate membrane. To silence lumican expression, each calvaria bone was infected with *lumican* shRNA CM or control shRNA CM in BGjb medium (Gibco) containing 100 U/mL penicillin, and 100 μg/mL streptomycin and 0.1% BSA in the presence of 8 μg/mL polybrene. After 3 days, each bone was cultured for an additional 10 days in a medium containing 50 μg/mL of insulin (Sigma-Aldrich). The bone then was fixed in 4% PFA for 24 h and decalcified in 0.5 M EDTA in PBS for 2 days. The calvaria specimens were embedded in paraffin, coronally sectioned at a thickness of 3 μm, and stained with H&E or toluidine blue O solution (Marino et al., 2016). Calvaria bone widths and the number of osteoblasts were assessed using the same methods described above.

### Statistical Analysis

Unless otherwise specified, all data are expressed as the mean ± standard error of the mean of at least three independent experiments relying on triplicate measurements. The significance of differences between two groups was assessed using the Mann–Whitney *U* test, whereas differences between 3 or more groups were tested using the analysis of variance with posthoc analysis via Tukey’s honest significance test (Kim et al., 2019). All statistical analyses were performed using SPSS version 18.0 (SPSS, Inc., Chicago, IL, United States). *P* < 0.05 was considered statistically significant.

## Results

### Increased Lumican Production During Myoblast Differentiation

**FIGURE 1:**
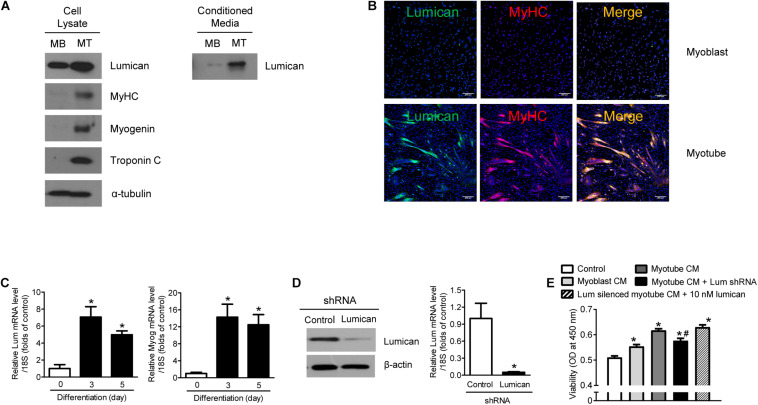
Increased lumican production during myoblast differentiation. **(A)** Mouse C2C12 myoblasts (MBs) were differentiated into myotubes (MTs) after treatment with 2% horse serum for 3 days. Lumican expression in lysates and conditioned media (CM) of MBs and MTs was determined by western blot analysis. **(B)** Immunocytochemistry images of lumican (green) and myosin heavy chain (MyHC; red) in MBs and MTs. **(C)** Quantitative RT-PCR analysis to determine *lumican* (*Lum*) and *myogenin* (*Myog*) expressions in C2C12 cells with or without treatment with 2% horse serum for the indicated days. **(D)** Western blot and quantitative RT-PCR analyses of lumican after infection with *lumican* shRNA CM for 6 h in C2C12 cells and differentiation into MTs after treatment with 2% horse serum for 3 days. **(E)** CCK-8 assay to assess the viability of preosteoblast MC3T3-E1 cells after exposure to 50% CM collected from MTs with or without *lumican* deletion. The 10 nM lumican was added in *lumican* silenced CM for the rescue experiment. Scale bar, 200 μm **(B)**. Data are presented as mean ± SEM. *P < 0.05 vs. control; ^#^*P* < 0.05 vs. myotube CM-treated group.

Western blot analysis reveals that lumican expression was higher in the cell lysates and CM of myotubes differentiated by 2% horse serum from mouse C2C12 myoblasts than in cell lysates and CM from undifferentiated cells (Figure 1A). Immunocytochemistry and qRT-PCR analyses further confirms that lumican expression was significantly higher in mature myotubes than in myoblasts (Figures 1B,C, respectively). A previous study has demonstrated that myotube CM markedly stimulates preosteoblast viability, compared to non-CM (i.e., control), and myoblast CM (Lee et al., 2019). To determine whether lumican acts as a myokine that affects bone metabolism, C2C12 myoblasts infected with *lumican* shRNA CM were differentiated into myotubes in the presence of 2% horse serum (Figure 1D), and their CM was collected. Interestingly, the stimulation of preosteoblast viability by myotube CM was markedly diminished by *lumican* silencing, and the addition of lumican to these CM rescued the reduced preosteoblast viability resulting from *lumican* knockdown (Figure 1E). Taken together, these results imply that lumican secreted from mature myotubes could function positively in osteoblast biology.

### Lumican Increases Bone Formation Through the Stimulation of Preosteoblast Viability and Differentiation

**FIGURE 2:**
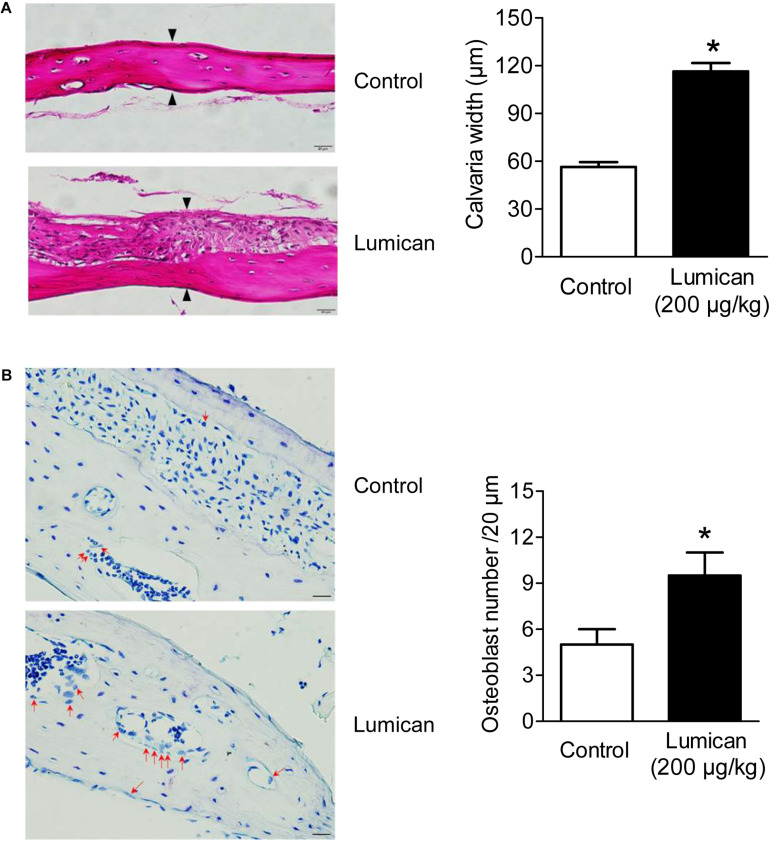
Increase in calvaria bone formation by lumican. Recombinant lumican was injected into the right side of the calvaria of 5-week-old male C57BL/6 mice for 4 weeks. PBS was injected into the left side of the calvaria as a negative control. **(A)** Hematoxylin and eosin staining was performed on the calvaria bone sections. Calvaria bone widths were quantitated by analyzing the average widths of five spots at the same interval of the midline between the sagittal suture and the site of muscle attachment. Black arrows indicate osteoid lines as reference for calvaria bone width measurements. **(B)** Toluidine blue O staining was also performed. Red arrows indicate osteoblasts, which are mono nuclear cuboidal cells on bone surface **(B)**. Scale bars, 20 μm **(A,B)**. Data are presented as mean ± SEM. *P < 0.05 vs. control.

To investigate the effects on *in vivo* bone formation, recombinant lumican and PBS were injected into the right and left sides of the mouse calvaria bone, respectively. Lumican treatment increased calvaria thickness by 2.1-fold, compared to the PBS-treated control (Figure 2A), and more osteoblasts were observed on the lumican-treated calvaria bone surface than in the control (Figure 2B).

**FIGURE 3:**
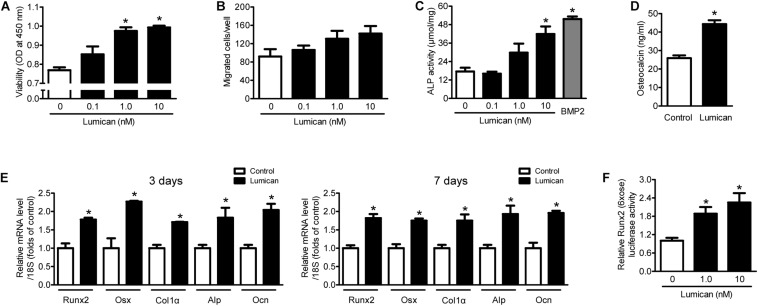
Lumican stimulates preosteoblast viability and differentiation. **(A)** The viability and **(B)** directional migration of preosteoblast MC3T3-E1 cells were assessed by CCK-8 assay and a Boyden chamber system, respectively, after exposure to the indicated concentrations of lumican for 24 h. **(C)** Alkaline phosphatase (ALP) activity and **(D)** osteocalcin secretion of MC3T3-E1 cells in osteogenic medium containing 50 μg/mL ascorbic acid and 10 mM β-glycerophosphate with or without 10 nM lumican for 7 days. ALP activity was normalized by total cellular protein amounts. **(E)** Quantitative RT-PCR expression analysis of osteoblast differentiation markers in MC3T3-E1 cells cultured in osteogenic medium with or without 10 nM lumican for 3 or 7 days. **(F)** Luciferase activity of *Runx2* after exposure to the indicated concentrations of lumican in osteogenic medium for 3 days. Data are presented as mean ± SEM. *P < 0.05 vs. control.

To supplement the mouse findings described above, we treated murine preosteoblast MC3T3-E1 cells with lumican *in vitro*. Lumican stimulated preosteoblast viability in a dose-dependent manner (Figure 3A), while preosteoblast migration changed little following lumican treatment (Figure 3B). Lumican markedly increased osteoblast differentiation, as determined by ALP activity (Figure 3C) and Ocn secretion (Figure 3D). Consistently, the expression levels of *Runx2* and *Osx*, and their target genes involved in osteoblast differentiation, including *Col1*α, *Alp*, and *Ocn*, were significantly increased by lumican (Figure 3E). Upregulation of *Runx2* was also confirmed by luciferase assays (Figure 3F). Collectively, these data indicate that lumican contributes to bone formation through the stimulation of preosteoblast viability and differentiation.

### The Effects of Lumican on Osteoblastogenesis Are Mediated by the Stimulation of the ERK Signal

**FIGURE 4:**
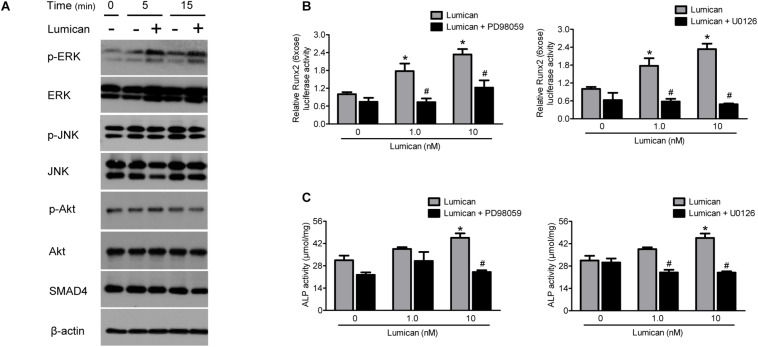
Effects of lumican on osteoblastogenesis are mediated by the simulation of ERK signal. **(A)** Western blot analysis to determine the activity of signals related to osteoblast differentiation after treatment with 10 nM lumican in MC3T3-E1 cells. **(B,C)** Murine preosteoblast MC3T3-E1 cells were pretreated with 20 μM PD98059 or 10 μM U0126, ERK inhibitors, followed by treatment with the indicated concentrations of lumican in the presence of 50 μg/mL ascorbic acid and 10 mM β-glycerophosphate. *Runx2* and ALP activities were assessed after 5 and 7 days, respectively. Data are presented as mean ± SEM. *P < 0.05 vs. control; ^#^*P* < 0.05 vs. lumican-treated group.

To determine the mechanism of action of lumican on osteoblasts, we focused on several signaling pathways related to osteoblast differentiation. Western blot analyses show that lumican increased the activity of ERK, but not those of JNK, Akt, and SMAD4 (Figure 4A). Importantly, pretreatment with the ERK inhibitors, PD98059 and U0126, almost completely reversed the lumican-mediated stimulation of *Runx2* and ALP activities (Figures 4B,C, respectively), indicating that ERK is a key signal regulating the effect of lumican on osteoblastogenesis.

### Integrin α2β1 Is the Major Receptor for Lumican in Osteoblasts

**FIGURE 5:**
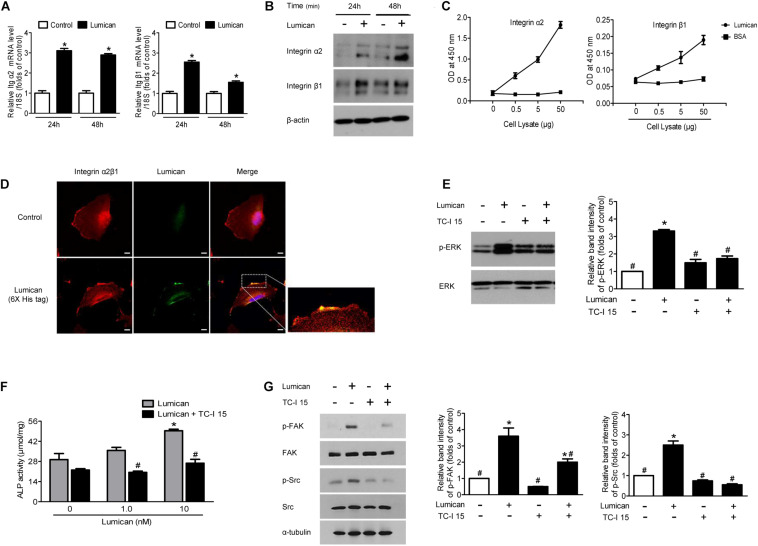
Integrin α2β1 is the major receptor for lumican on osteoblasts. **(A,B)** Quantitative RT-PCR and western blot analyses to determine the activity of integrin α2 and β1 after treatment with 10 nM lumican in MC3T3-E1 cells, respectively. **(C)** Interaction of lumican with integrin α2 and β1 in MC3T3-E1 cells using a binding ELSIA assay. Different amounts of cell lysates were incubated in lumican- or BSA-coated wells, followed by the determination of the amounts of integrin α2 and β1 by ELISA. **(D)** Immunofluorescence images of integrin α2β1 (red) and lumican (green) in human fetal osteoblasts after treatment with 40 nM 6X His-tagged lumican for 30 min. **(E)** Western blot analysis to determine the phosphorylation of ERK after pretreatment with 1 μM TC-I 15, an integrin α2β1 inhibitor, followed by treatment with 10 nM lumican for 5 min. **(F)** MC3T3-E1 cells were pretreated with 1 μM TC-I 15, followed by treatment with the indicated concentrations of lumican in the presence of 50 μg/mL ascorbic acid and 10 mM β-glycerophosphate for 3 days, and ALP activity was assessed. **(G)** Western blot analysis to determine the phosphorylation of FAK and Src after pretreatment with 1 μM TC-I 15, followed by treatment with 10 nM lumican for 5 min. Scale bar, 10 μm **(D)**. Data are presented as mean ± SEM. *P < 0.05 vs. control; ^#^*P* < 0.05 vs. lumican-treated group.

Transmembrane integrin α2β1 is known to be involved in the survival and differentiation of osteoblast lineages (Popov et al., 2011; Shih et al., 2011) as well as in the action of lumican in other cell types (Seomun and Joo, 2008; Brezillon et al., 2013). We thus investigated whether the protective role of lumican on osteoblasts is mediated by integrin α2β1. Quantitative RT-PCR and western blot analyses show that integrin α2 and β1 were expressed in MC3T3-E1 cells and lumican treatment augmented their levels of expression (Figures 5A,B, respectively). A binding affinity experiment using ELISA shows that the amount of lumican-associated integrin α2 and β1 increased as the amount of osteoblast lysate increased (Figure 5C), indicating that lumican directly associates with integrin α2β1 in osteoblasts. Immunofluorescence analysis also shows the interaction between lumican and integrin α2β1 in osteoblasts (Figure 5D). In support of these findings, pretreatment of osteoblasts with an integrin α2β1 inhibitor, TC-I 15, attenuated the phosphorylation of ERK (Figure 5E), and blocked lumican-mediated stimulation of ALP activity (Figure 5F). The FAK/Src signaling is well known for the critical mediator of integrin, leading to the activation of ERK (Mitra and Schlaepfer, 2006; Bouchard et al., 2008). Importantly, pretreatment of osteoblasts with TC-I 15 reversed the lumican-mediated stimulation of FAK and Src (Figure 5G), further supporting that lumican exerts function in osteoblast via integrin α2β1 and downstream ERK pathway.

### Lumican Deletion Reduces Calvaria Bone Formation

**FIGURE 6:**
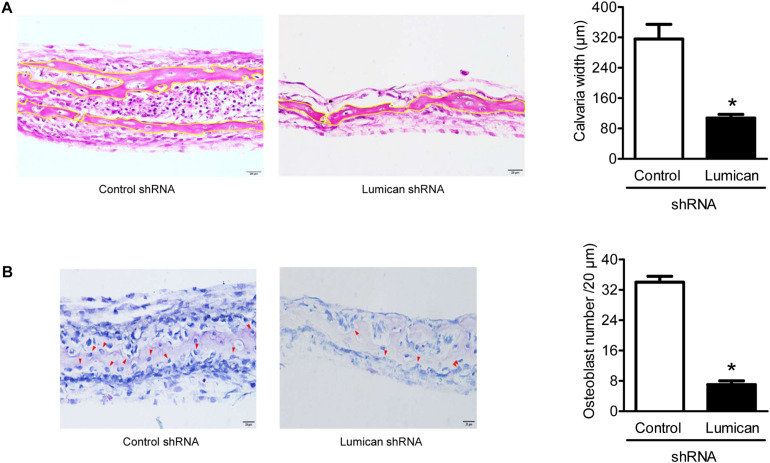
Lumican deletion reduces calvaria bone formation. The calvaria bones dissected from timed-pregnant mice at E21.0 were infected with *lumican* shRNA CM in BGjb medium (containing 100 U/mL penicillin) as well as 100 μg/mL streptomycin and 0.1% BSA (1:1 ratio) for 3 days and additionally cultured in a medium containing 50 μg/ml insulin for 10 days. **(A)** Hematoxylin and eosin staining was performed on the specimens, and calvaria bone widths were quantitated by analyzing the average of five spots. **(B)** Toluidine blue O (blue) staining was performed on the specimens, and the number of osteoblasts (red arrows) was counted. Scale bars, 20 μm **(A, B)**. Data are presented as mean ± SEM. *P < 0.05 vs. control.

To confirm the importance of lumican in bone formation, we adopted the models of *ex vivo* explant cultures. The dissected calvaria bone from time-pregnant mice at E21.0 were infected with *lumican* shRNA CM, and then incubated in organ culture media. Compared to the control, calvaria thickness and osteoblast numbers were significantly decreased by the silencing of *lumican* (Figures 6A,B, respectively), supporting the view that lumican acts anabolically in bone metabolism.

## Discussion

We have shown here that lumican secreted from mature myotubes stimulates bone formation by increasing preosteoblast viability and differentiation, and these effects are likely mediated by integrin α2β1 and the downstream ERK signaling in osteoblasts. These findings suggest that lumican could be a myokine that, at least in part, mediates biochemical bone-muscle interactions and be a potential therapeutic target for metabolic bone diseases.

With a mass of approximately 40 kDa, lumican belongs to the family of small leucine-rich repeat proteoglycans (SLRPs; Blochberger et al., 1992). Originally identified as a major proteoglycan of the cornea (Hassell et al., 1980), lumican is now known to be widely expressed in different tissues, including skin, lung, kidney, breast, colon, artery, and cartilage (Dolhnikoff et al., 1998; Leygue et al., 1998; Schaefer et al., 2000; Nikitovic et al., 2008b). In addition to its well-known role in the regulation of the structural organization of these tissues, evidence now indicates that lumican is involved in diverse biological functions such as cell adhesion, migration, proliferation, and differentiation (D’Onofrio et al., 2008; Nikitovic et al., 2008a,b). Interestingly, previous proteomic analyses described lumican as a novel muscle-secreted factor (Norheim et al., 2011). The fitness benefits of the secretion of anabolic factors from muscle to bone is currently speculative, thus we tested whether lumican has protective effects in terms of bone metabolism. Our *in vitro* and animal experiments demonstrate that muscle-derived lumican directly stimulates osteoblastogenesis and bone formation.

Among the various transcriptional factors controlling osteogenesis, *Runx2* is regarded as a master switch regulating the expression of osteoblast-specific genes including *Col1*α, *Alp*, and *Ocn* (Ducy et al., 1999). Thus, *Runx2*-deficient mice lack osteoblasts and fully mineralized bone matrix (Otto et al., 1997). Because of the critical role RUNX2 plays in osteoblastogenesis, this protein is tightly regulated by various cellular factors (Ge et al., 2012). In investigating the effects of lumican on RUNX2-related signals, we showed that lumican induces ERK activation in osteoblasts, which is consistent with observations in other cell types (Seomun and Joo, 2008). Moreover, ERK inhibitors attenuate lumican-stimulated *Runx2* and ALP activities. Collectively, these results indicate that lumican-stimulated osteoblast differentiation may be regulated by the ERK pathway.

To determine the receptor mediating the actions of lumican on osteoblasts, we focused on integrin α2β1, based on the existing evidence. In particular, experimental evidence suggests that integrin α2β1 participates in the survival and differentiation of osteoblast lineages through the activation of ERK (Popov et al., 2011; Shih et al., 2011). In the present study, an integrin α2β1 inhibitor reduced lumican-induced ERK and ALP activities, suggesting that integrin α2β1 is the major receptor for lumican in osteoblasts.

Although we focused on the direct interaction of lumican with transmembrane integrin α2β1 receptor and the resultant downstream ERK activation in osteoblasts, previous study reported that U0126, the ERK inhibitor, suppressed the integrin β1 expression induced by lumican in corneal epithelial cells (Seomun and Joo, 2008). These results suggest the other scenario where the expression of integrin β1 is regulated by ERK phosphorylation in response to lumican. Therefore, the interplay among lumican, integrin, and ERK signal would be interesting topic to further understand the action mechanism of lumican on bone metabolism.

In summary, lumican is expressed in both the cell lysates and CM of myotubes. The known anabolic effects of myotube CM on bone is reduced when lumican expression in the CM is silenced. In addition, the results of additional experiments revealed that lumican could directly contribute to bone formation through the positive effects on osteoblast biology. More extensive *in vivo* and human studies are needed to confirm these experimental findings and determine their clinical implications.

## Data Availability Statement

The datasets used and/or analyzed during the current study are available from the corresponding author.

## Ethics Statement

The animal study was reviewed and approved by the Institutional Animal Care and Use Committee of the Asan Institute for Life Sciences.

## Author Contributions

J-MK and B-JK: conceptualization. JL, SP, DK, and B-JK: data acquisition. JL, SP, DK, and B-JK: data analysis and interpretation. JL, SL, and B-JK: drafting of the manuscript. All authors have read and agreed to the final version of the manuscript.

## Conflict of Interest

The authors declare that the research was conducted in the absence of any commercial or financial relationships that could be construed as a potential conflict of interest.
